# Role of Macroautophagy in Nutrient Homeostasis During Fungal Development and Pathogenesis

**DOI:** 10.3390/cells1030449

**Published:** 2012-08-02

**Authors:** Yizhen Deng, Ziwei Qu, Naweed I. Naqvi

**Affiliations:** Temasek Life Sciences Laboratory, Department of Biological Sciences, 1 Research Link, National University of Singapore, Singapore 117604, Singapore; Email: yizhen@tll.org.sg (Y.D.); ziwei@tll.org.sg (Z.Q.)

**Keywords:** autophagy, degradation, metabolism, fungi, pathogen, ROS

## Abstract

Macroautophagy is a non-selective, bulk degradation process conserved in eukaryotes. Response to starvation stress and/or regulation of nutrient breakdown/utilization is the major intracellular function of macroautophagy. Recent studies have revealed requirement for autophagy in diverse functions such as nutrient homeostasis, organelle degradation and programmed cell death in filamentous fungal pathogens, for proper morphogenesis and differentiation during critical steps of infection. In this review, we aim to summarize the physiological functions of autophagy in fungal virulence, with an emphasis on nutrient homeostasis in opportunistic human fungal pathogens and in the rice-blast fungus, *Magnaporthe oryzae*. We briefly summarize the role of autophagy on the host side: for resistance to, or subversion by, the pathogens.

## 1. Introduction

Autophagy, comprising selective or non-selective types, utilizes the vacuolar/lysosomal system for bulk degradation of certain organelles, proteins and/or membranes in eukaryotic cells. Molecular basis of autophagy was established through identification of 35 *ATG* (AuTophaGy related) genes [1,2], in the past decade of intensive research in yeasts and mammalian cells. Some Atg proteins are involved in common steps for both non-selective and selective autophagy, such as Atg1–10, Atg12–18, and Atg22 [3,4,5,6,7,8,9,10,11,12,13,14]. Others are involved in selective autophagy, including Atg11, Atg19–21, Atg23–24, Atg27 and Atg34 for the CVT (Cytosol-to-Vacuole Targeting, selectively delivering two vacuolar enzymes) pathway [15,16,17,18,19,20]; Atg25, Atg26, Atg28 and Atg30 for pexophagy (autophagic degradation of peroxisomes, the membrane-bound organelles responsible for lipid metabolism) [21,22,23,24]; Atg32–33 and 35 for mitophagy (autophagic degradation of mitochondria) [1,25,26]. Autophagy plays important roles in multiple aspects of physiological (e.g., morphogenesis, stress response and programmed cell death) [27,28,29,30,31,32,33] or pathological (cancer or neurodegenerative disease etc) [34,35,36,37] processes. 

As plant or animal pathogens, filamentous fungi have great impact on agriculture and health care. Research on fungal development and differentiation provide potential targets for bio-control and/or fungicide exploitation. However, studies on the role of autophagy in pathogenic yeasts or fungi have just recently begun to emerge, and are still less advanced [38,39]. At present, available methods on autophagy research in filamentous fungi include: (1) genetic study with autophagy-deficient mutants, and induction or inhibition of autophagy by established chemicals such as Rapamycin, Wortmannin, Vinblastine or 3-methyladenine; (2) morphological characterization of autophagosomes and/or autophagic bodies, autophagic vacuoles, with Transmission Electron Microscopy (TEM) or epifluorescence or confocal microscopy; (3) specific biochemical assays, RTPCR(Reversed Transcriptase PCR) or western blotting [40,41].

Most pathogenic fungi undergo a life cycle composed of two important stages: asexual or sexual spore formation and invasive growth in the host tissues. During sporulation, abundant energy and/or materials requirements need to be fulfilled for proper cellular structure build-up and morphogenesis. For invasive growth in the host tissues, fungal pathogens are exposed to an inhospitable environment, with nutrient constraints and oxidative stress as a host defense mechanism. Autophagy may act in both stages in terms of nutrient homeostasis and/or anti-oxidative response, in favor of pathogens for successful sporulation and infection. In this review, we will discuss the multiple functions of autophagy in fungal development and pathogenesis, with an emphasis on carbon homeostasis through autophagy pathway for *Magnaporthe oryzae* asexual sporulation and pathogenic development. Ultimately, we hope to summarize the known substrates from cell constituents, including carbohydrate and nitrogen sources, for autophagy degradation during fungal differentiation, and how such self-eating behavior contributes to pathogen adaptation, reproduction and/or pathogenesis.

## 2. Induction and Requirement of Autophagy in Development and/or Pathogenesis of Filamentous Fungi

Autophagy induction can be directly visualized by fluorescent marker tagged Atg8, which localizes to autophagosome(s) and is internalized in vacuole(s) after fusion between autophagosomes and vacuoles [41,42]. Alternatively, transcriptional up-regulation of relevant *ATG* genes was also accepted as an indicator of autophagy induction [41]. Natural induction of autophagy has been reported in various fungal organisms, indicative of a functional requirement, during sporulation or infection. Examples are as follows: upon photo-induction of conidiation, autophagosomes /autophagic vacuoles marked by RFP-Atg8 were abundant in aerial hyphae, stalk and conidiophore of *M. oryzae* [43]. During the differentiation of conidiophores and conidial germination of *Aspergillus oryzae*, DsRed2-AoAtg8 or enhanced GFP (Green Fluorescent protein) tagged AoAtg8 accumulated in the vacuole [44]. Atg8 expression was up-regulated in *Cryptococcus neoformans* during a human brain infection [45], indicating autophagy induction. Transcription of *ATG3* and *ATG9* was up-regulated during murine macrophage infection by *C. neoformans* [46]. Atg7 transcription was up-regulated at late stage of sexual development of *Sordaria macrospora* [47].

In filamentous/model fungi, the following ATG genes were shown to be conserved with their orthologs in yeasts or animals: *ATG1*, *2*, *4*, *5*, *7*, *8*, *9*, *15*, *17*, and *18* [43,44,45,46,47,48,49,50,51,52,53,54,55,56,57,58,59,60,61,62,63]. *VPS34* encodes a phosphatidylinositol 3-kinase (PtdIns3K) that induces autophagy, and is also conserved in fungi [45]. Functional requirement of autophagy for fungal development and pathogenesis was investigated by characterization of autophagy-deficient mutants. Such work has been carried out, and reported in diverse fungal systems. Autophagy-deficient fungal mutants showed defects in conidiation and/or virulence. Examples include: *Fusarium graminearum atg15*Δ [48] and *atg8*Δ [49], *Trichoderma reesei atg5*Δ [50], *Aspergillus fumigatus atg1*Δ [51,52], *Colletotrichum lindemuthianum clk1* (a homolog of *atg1*) deletion mutant [53], *M. oryzae atg1*Δ [54], *atg2*Δ, *atg4*Δ, *atg5*Δ, *atg9*Δ, *atg18*Δ [55], or *atg8*Δ [43,56] , *A. oryzae atg8*Δ [44], and *C. neoformans vps34*Δ mutants [45]. Similarly, *Ustilago maydis atg8*Δ and *atg1*Δ mutants showed reduced teliospore formation and virulence [57]. Appressorium formation was compromised in *Colletotrichum orbiculare atg8*Δ or *atg26*Δ [58,59]. *S. macrospora atg7* RNAi mutant showed defective fruiting-body development, and autophagy is required for fungal viability [47]. *Candida glabrata atg17*Δ showed reduced survival in host tissues [60]. Interestingly, although non-selective autophagy was shown to be essential for *M. oryzae* pathogenesis [43,54,55,56], selective subtypes of autophagy, including pexophagy, seem dispensable [61]. Different from *M. oryzae*, pexophagy is necessary for *C. orbiculare* pathogenesis [58]. This suggests that for fungal pathogenicity, organelle turnover through autophagy may not be as vital as bulk degradation of cellular constituents. Among various pathogenic/model fungi investigated, only *Candida albicans* seems to be an exception. Autophagy plays little or no physiological role in the differentiation or pathogenicity, although specific defects in autophagy and the Cvt pathways were seen in the *atg9*Δ mutant in *C. albicans* [62,63].

Despite a large number of reports on the requirement of autophagy in fungal differentiation and pathogenesis, the actual mechanistic role(s) of autophagy remain largely unknown in abovementioned fungi. Starvation was the first stimulus found to induce autophagy [64] and also one of the major sporulation inducers in filamentous fungi [65]. On the other hand, nutrient availability is generally limited in the host, so that the ability of the fungal pathogen to trigger autophagy might be associated with an overall capacity to cope with nutrient-deficient environment. Based on such knowledge, it makes sense to speculate that autophagy-mediated intracellular recycling may guarantee proper sporulation and/or invasive growth in a nutrient-deprived environment. Indeed, studies on *M. oryzae*, *A.* *fumigatus* and *A.* *oryzae* have shown that autophagic/vacuolar degradation facilitates utilization of cellular storages of carbohydrate (glycogen or lipid droplets) [43,48,49,54,56,57], nitrogen sources [52], or even the nuclei [44], as a source of nutrients. Table 1 summarizes the diverse functions of autophagy in filamentous fungi.

**Table 1:** Summary of autophagy functions documented in model fungi.

Fungus	Host	Mutants analyzed	Phenotypic defects	Deduced Autophagy Function	References
*Magnaporthe oryzae*	Rice,Barley	*atg1*Δ, *atg2*Δ, *atg4*Δ, *atg5*Δ, *atg8*Δ, *atg9*Δ, *atg18*Δ	Reduced conidiation; non-pathogenic	Glycogen breakdown; nuclear degradation; Turgor; lipid droplet degradation; autophagy cell death	[43,54,55,56,61]
*Aspergillus oryzae*	N.A.	*atg1*Δ	Reduced conidiation	Nuclear degradation	[44]
*Ustilago maydis*	Corn	*atg8*Δ	Reduced teliospores production and pathogenicity	Possibly glycogen metabolism	[57]
*Cryptococcus neoformans*	Human	*vps34*Δ,*ATG8-RNAi*	Reduced virulence	Likely nutrient homeostasis	[45,46]
*Fusarium graminearum*	Rice, Barley Wheat	*atg15*Δ,*atg8*Δ	Reduced conidiation; non-pathogenic	Lipid droplet turnover; likely glycogen breakdown	[48,49]
*Trichoderma reesei*	N.A.	*atg5*Δ	Reduced conidiation	Not clear	[50]
*Aspergillus* *fumigatus*	Human Murine	*atg1*Δ	Reduced conidiation	Nitrogen metabolism; metal metabolism	[51,52]
*Colletotrichum lindemuthianum*	Beans	*clk1*Δ (*atg1*Δ)	Reduced pathogenicity	Not clear	[53]
*Colletotrichum orbiculare*	Cucumber	*atg8*Δ	No appressorium formation (non-pathogenic)	Not clear	[58,59]
*Sordaria macrospora*	N.A.	*atg7* RNAi	Aberrant fruit-body formation	Not clear	[47]
*Candida glabrata*	Human	*atg17*Δ	Reduced survival in host	Likely ROS and / or starvation resistance	[60]
*Candida albicans*	Human	*atg9*Δ	No defects	Not clear	[62,63]

## 3. Autophagy-Dependent Nutrient Homeostasis in Fungal Sporulation and Pathogenesis

During fungal conidiation / sporulation and infection, autophagic degradation may produce abundant nutrients and small molecules for energy source or materials to build up new intracellular structures, or as an adaption to adverse host environment. This is particularly important, because nutrient deprivation may commonly occur during such growth phase of development and morphogenesis. Taking *M. oryzae* as an example, we discuss nutrient homeostasis via autophagy during fungal conidiation and pathogenesis in this section.

**Figure 1:**
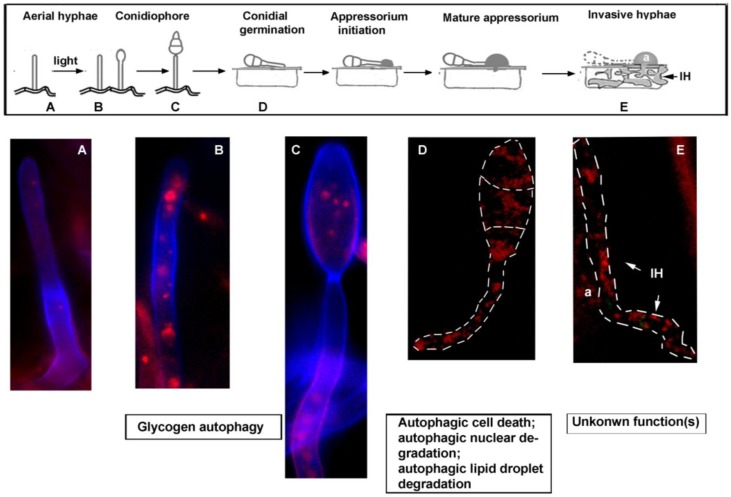
Schematic diagram of *M. oryzae* pathogenic life cycle, and natural induction of autophagy. Schematic representation of the pathogenic life cycle of *M. oryzae* (boxed), with corresponding steps assessed for autophagy (RFP-Atg8) induction depicted in (**A–E**). Basal level of RFP-Atg8 is undetectable in the aerial hyphae grown in the dark (**A**). Upon photo-induction, RFP-Atg8 is naturally induced in the aerial hyphae (**B**), as well as in the conidiophore (**C**). For (**A**)–(**C**), *Magnaporthe* strain expressing RFP-Atg8 was grown on PA (prune agar) medium, co-stained with Calcofluor White and analysed by confocal microscopy. RFP-Atg8 was also naturally induced during conidial germination (**D**) and in invasive hypha (**E**). For (**D**)**–**(**E**), dashed lines were used to delineate the outline of the analyzed fungal structures. a, appressorium; IH, invasive hypha. Arrows in (**E**) mark primary invasive hypha (36–40 hpi (hours post inoculation)).

*M. oryzae* is a filamentous ascomycete that causes a devastating blast disease in rice [66]. It produces asexual spores called conidia upon photo-induction. A mature conidium is pyriform and is composed of three cells [67]. *M. oryzae* conidia are dispersed by air, and responsible for the spread of blast disease [68]. Upon germination, a conidium differentiates into a dome-shaped structure, called appressorium, at the tip of the germ tube. Appressorium facilitates entry and colonization of the host by generation of a high hydrodynamic turgor for mechanical breach of host surface [69,70,71]. Once the fungus successfully colonizes its host, it initiates conidiation within and continues subsequent rounds of pathogenic life cycle [72]. A simplified schematic diagram showing *M. oryzae* pathogenic life cycle is included in Figure 1.

The *atg8*Δ mutant of *M. oryzae* showed significant reduction in conidiation, which could be restored by addition of alternative carbon sources, glucose or sucrose, or glucose-6-phosphate [43,54,56]. A proteomics study showed that glycogen phosphorylase was differentially expressed in the *atg8*Δ mutant and detailed analysis on glycogen catabolism established a role for autophagy in carbon source utilization during *M. oryzae* conidiation [43,73]. Conidiation defects in *M. oryzae atg8*Δ mutant could not be restored by addition of nitrogen sources (ammonium chloride or sodium nitrate) [73], indicating that *M. oryzae* conidiation may be independent of autophagic nitrogen catabolism. Autophagy transports cytosolic glycogen into vacuoles for bulk degradation and thus production of great amount of glucose as energy source or cell wall synthesis material, or glucose-6-phosphate as messenger molecule. Vacuolar glycogen degradation was shown to be important for *M. oryzae* conidiation and is autophagy-dependent [73]. Methods for assessing total glycogen level in *M. oryzae* include enzymatic hydrolysis and spectrophotometry-based glycogen / starch quantification, and semi-quantitative assay by iodine staining [43]. However, neither of these two assays specifies the subcellular localization (cytosolic or vacuolar) of glycogen contents. TEM examination of glycogen particles, judged by the size, is well established in animal and human cells [74,75,76] but not reported in *M. oryzae* or other fungi.

On the other hand, autophagy-deficient mutants were non-pathogenic. The *atg8*Δ showed defects in appressoria-mediated host penetration. The requirement of autophagy during appressorial development seems irrelevant to glycogen hydrolysis, but probably relevant for nuclear and/or lipid droplet degradation, and/or autophagic cell death [54,56]. A recent publication showed that macroautophagy, but not Piecemeal Microautophagy of the Nucleus (PMN), is responsible for nuclear degradation necessary for *M. oryzae* infection [77]. Furthermore, ER (endoplasmic reticulum) degeneration and vacuole degeneration within conidia during this process also seem to be dependent on non-selective macroautophagy [77]. By using RFP-Atg8 as an established marker for autophagy, we observed natural induction of autophagy during multiple stages of *M. oryzae* pathogenic life cycle (Figure 1). However, the specific role(s) of autophagy at different stages of *Magnaporthe* development are not fully understood, but are suggestive of both cell survival and cell death functions [54,56,77].

Other studies indicative of importance of nutrient homeostasis for *M. oryzae* conidiation and/or pathogenesis include the creation and characterization of the *snf1*Δ mutant [78] and the *tps1*Δ mutant [79]. Snf1 was first examined in *Saccharomyces cerevisiae* as a gene required for the de-repression of catabolite-repressed genes when glucose becomes limiting [80]. In *S. cerevisiae*, a high level of glucose in the growth medium results in the repression of gene expression for gluconeogenesis, respiration, and use of alternative-carbon-sources as a mechanism for efficient energy utilization [80]. Snf1 was also shown to positively regulate both glycogen synthesis (in cytosol) and autophagy [81]. *M. oryzae snf1*Δ mutant displayed reduced conidiation and abnormal conidial morphology [78]. However, autophagy was not assessed in the *snf1*Δ mutant. Interestingly, glucose-6-phosphate metabolism regulated by Tps1, trehalose-6-phosphate (T6P) synthase, is relevant with nitrogen source utilization [79]. Tps1 is a central regulator for integration of carbon and nitrogen metabolism, and its regulatory functions are associated with binding of G6P, but independent of Tps1 catalytic activity [79]. Therefore nitrogen metabolism may still be important for *M. oryzae* asexual differentiation in an indirect and autophagy-independent manner.

Besides *M. oryzae*, autophagy-assisted carbon utilization was also reported in *F. graminearum* [49]. Autophagy dependent lipid utilization, lipophagy, was suggested in *F. graminearum* [48] and *U. maydis* [57], for conidiation / teliospore formation and plant colonization. Similarly, autophagy-mediated nutrient catabolism for the efficiency of asexual sporulation was demonstrated in *A. oryzae*. One possible function of autophagy in *A. oryzae* is to mediate uptake and degradation of whole nuclei, likely as a source for nutrients to support mycelial growth in order to counteract starvation [82].

In *Fusarium oxysporum*, indirect evidence suggests that autophagy may be induced as a consequence of disruption of global nitrogen regulation and is important for fungal survival [83]. However, direct connection between autophagy and fungal virulence remains to be explored. In *Moniliophthora perniciosa*, carbon source has a significant influence on cellular sensitivity to oxidative stress by inducing autophagy as a response of nutrient constraint [84]. Such autophagy-dependent nutrient catabolism and ROS resistance may mimic *in planta* growth and thus essential for pathogenesis of *M. perniciosa*.

In *Aspergillus nidulans* carbon-starvation-triggered autolysis is coupled with sporulation initiation, and supplying nutrients for sporulation, when no other sources of nutrients are available [85]. Autophagy has been shown to precede autolysis [86], and may possibly play a role in autolysis and/or sporulation in *A. nidulans*.

Thus, based on the proposed function of autophagy for glycogen hydrolysis in *M. oryzae* conidiation, and related phenomena observed in other model fungi, we believe that autophagy-assisted nutrient catabolism/recycling in concert with cellular re-modeling or morphogenesis, likely represents a common scheme in fungal growth, differentiation and/or pathogenesis.

## 4. Autophagy in Opportunistic Human Fungal Pathogens

Autophagy function was also reported as a virulence factor in human opportunistic fungal pathogens. But dependence on autophagy for infection appears evolutionarily divergent among the four opportunistic human fungal pathogens discussed here. In this section, we wish to summarize the studies on autophagy in pathogenic fungi/yeast that infect humans. Autophagy is required for successful infection by *C. neoformans*. Autophagy genes *ATG3*, *ATG8* and *ATG9* were shown to be transcriptionally up-regulated in *C. neoformans* during infection of murine or human cell lines [45,46]. Vps34, an upstream inducer of autophagy, was also shown to be essential for pathogenesis in *C. neoformans* [45]. A *vps34*Δ mutant showed impaired autophagy, reduced viability under starvation, and fast clearance from the infected host tissues, which was similar to the autophagy-deficient CnATG8 RNAi strain [45]. Autophagy was thus proposed to play a role in the adaptation to nutrient starvation and acts as a survival mechanism / virulence contributor for Cryptococcal species [45].

Autophagy is also a likely virulence factor for *C. glabrata* [60]. The *cgatg17*Δ mutant displayed lower survival rate after phagocytosis, probably due to loss of autophagy-mediated nutrient utilization under the sustained carbon starvation within host macrophages [60]. *C. glabrata* likely depends on autophagy, which appears to be an important virulence determinant, for survival and intra-host viability through mobilizing intracellular nutrient resources during pathogenesis [60].

Different from *C. neoformans* and *C. glabrata*, engulfed *C. albicans* cells induce many genes involved in non-fermentative carbon metabolism to cope with nutrient deprivation [87], and a catalase activity to decompose oxidants produced by the host [87,88]. Therefore, based on current knowledge autophagy seems to play limited or no role in *C. albicans* infection. In *A. fumigatus*, autophagy is dispensable for virulence [89] but required for sporulation [52]. An *Afatg1* mutant showed compromised sporulation, which could be restored by supplementation of exogenous nitrogen source (ammonium tartrate) [52]. The reason behind variant requirements for autophagy in these four pathogens was discussed systematically in a recent review [89], and was indicated to be based on the difference in the aspects such as the host infection niche and the evolutionary pressures faced by the pathogen species [89], the host signals in response to the pathogen [89], and molecular basis of virulence for each pathogen [90].

## 5. Host Autophagy as a Defense Mechanism or Facilitator of Infection

On the host side, autophagy acts as a front line innate immune response against invasive microbes [91]. Autophagy is induced upon pathogen invasion, and has a role in elimination of intracellular pathogens, mostly as bacteria, virus or parasites in literature [91,92]. Besides capture and degradation of invading microbes, autophagy may also collaborate with other intracellular immune systems against pathogen infection. A recent finding suggests that autophagy components are involved in ROS (reactive oxygen species) production in response to bacterial infection [93]. Reports on autophagy-mediated antifungal activity are limited [94,95]. Autophagy can regulate programmed cell death as plant defense against biotrophic pathogens [96]. In Arabidopsis autophagy cooperates with other plant defense pathways in the regulation of plant innate immunity to necrotrophic pathogens *Botrytis cinerea* [97]. ROS production is an important feature of plant disease resistance. We recently identified a sorting nexin in *M. oryzae* that mediates anti-oxidative response during fungal invasive growth [98]. In this context, it would be interesting to investigate the possible role of autophagy in regulating redox homeostasis in rice (*M. oryzae* host) during fungal infections.

In contrast, *C. neoformans* was recently reported to exploit host autophagy proteins and other intracellular trafficking and signaling molecules to establish a replicative niche [99]. Pharmacological inhibition of autophagy and/or PI3-kinase activity suppressed *C. neoformans* infection [99]. In this case, host Atg proteins mediate *C. neoformans* intracellular trafficking and replication. Despite serving an anti-pathogen function, host autophagy can also be manipulated by the pathogen to favor its replication and spread during infections.

## 6. Conclusions

Despite abundant knowledge on molecular basis of autophagy, following two decades of intense studies in *S. cerevisiae* and other model systems, our knowledge about specific functions of autophagy in host-pathogen interaction is still quite limited. Considering the importance of filamentous fungi in medicine, agriculture and science, detailed knowledge of autophagy in filamentous fungi and their hosts may not only help in a better understanding of the host-pathogen interaction, but also provide a more rational basis for the design of antifungal drugs.

Autophagy studies in filamentous fungi have concentrated on macroautophagy, induced by nutrient depletion and/or oxidative stress conditions, which excellently mimic the environment that invasive pathogens may encounter within the host tissues. Autophagy helps the pathogenic fungi to adapt to the adverse conditions by regulating utilization of their own cellular storage of nutrients, and thus better infect and colonize the host. Host cells can also induce autophagy as a defense mechanism against invading fungi, to either eliminate the intracellular pathogen or accelerate programmed cell death (probably through triggering ROS production as a cell death signal) at the site of infection as a hypersensitive reaction (HR). However, host autophagy machinery could also be exploited by the invading pathogen to facilitate its survival and/or spread. This needs to be taken into consideration for pharmaceutical development using Atg proteins as potential antifungal targets. 

Future studies would be expected to further our knowledge on the regulation of autophagy during fungal differentiation. Particularly, how is the nutritional condition sensed and conveyed through Snf1 and /or Tps1 enzymatic activity, leading to autophagy induction? How is autophagy regulated in response to oxidative stress imposed by the host, and more importantly, how exactly does autophagy contribute to ROS scavenging? On the host side, how are the dual functions of autophagy as microbial degradation and as ROS production triggered, integrated, and spatiotemporally regulated? Further studies are certainly needed on the roles of autophagy in hosts and their cognate fungal pathogens to specifically address such pertinent questions in fungal diseases in plants and humans.
